# Long-Term Stability of Cyclosporine Blood Concentrations Assessed by Patient-Based Percentiles over 20 Years

**DOI:** 10.3390/pharmaceutics18070787

**Published:** 2026-06-27

**Authors:** Anders Larsson, Mathias Karlsson, Anna-Karin Hamberg

**Affiliations:** Department of Medical Sciences, Clinical Chemistry and Pharmacology, Uppsala University, SE-751 85 Uppsala, Sweden; mathias.karlsson@capitainer.se (M.K.); anna-karin.hamberg@akademiska.se (A.-K.H.)

**Keywords:** cyclosporine, patient median, therapeutic drug monitoring, immunosuppressive, transplantation, patient-based quality control (PBQC), pharmacokinetics, clinical laboratory quality assurance

## Abstract

**Background/Objectives:** Therapeutic drug monitoring (TDM) of cyclosporine is essential due to its narrow therapeutic index and pronounced pharmacokinetic variability. Long-term surveillance of patient results may provide insight into analytical stability and clinical practice patterns beyond conventional quality control approaches. **Methods:** This retrospective observational study included 48,835 routine whole blood cyclosporine concentrations analyzed at a tertiary university hospital laboratory between January 2006 and December 2025. Yearly patient percentiles (10th, 25th, 50th, 75th and 90th percentiles) were calculated to assess longitudinal trends, variability, and potential effects of analytical platform transitions. Results were analyzed overall and by sex. **Results:** The yearly number of reported cyclosporine results declined modestly over the study period. The overall median cyclosporine concentration was 134.4 µg/L, with negligible differences between female and male patients. The 10th, 25th, and 50th percentiles remained highly stable across the 20-year period, with coefficients of variation between 6.1% and 6.8%. Upper percentiles exhibited greater variability, but the total coefficient of variation for the 90th percentile remained below 8%. No systematic shifts associated with analytical platform transitions were observed. **Conclusions:** Long-term patient median and percentile analysis demonstrated remarkable temporal stability of cyclosporine concentrations over two decades, despite changes in analytical platforms and clinical practice. Continuous monitoring of patient medians and percentiles may serve as a valuable complementary quality indicator, particularly for assays with limited commutable quality control materials.

## 1. Introduction

Cyclosporine is a calcineurin inhibitor and an immunosuppressive medication widely used to prevent organ rejection following solid organ transplantation, including kidney, liver, and heart transplantation [1,2]. In addition, cyclosporine is used in selected autoimmune and inflammatory disorders such as severe rheumatoid arthritis, psoriasis, and aplastic anemia [3,4]. Its immunosuppressive effect is predominantly mediated through inhibition of T-lymphocyte activation, a central process in adaptive immune responses and allograft rejection [5].

Cyclosporine exerts its pharmacological action by binding to cyclophilin, forming a drug–protein complex that inhibits the phosphatase activity of calcineurin [6,7]. This inhibition prevents the dephosphorylation and nuclear translocation of the nuclear factor of activated T cells (NFAT), leading to reduced transcription of interleukin-2 (IL-2) and other cytokines essential for T-cell proliferation and activation. Through this mechanism, cyclosporine effectively suppresses cell-mediated immune responses.

Because cyclosporine has a narrow therapeutic index, careful monitoring of blood concentrations is essential to balance the risk of toxicity against insufficient immunosuppression. Excessive cyclosporine exposure is associated with adverse effects such as nephrotoxicity, hypertension, neurotoxicity, and dyslipidemia, whereas subtherapeutic levels increase the risk of acute and chronic graft rejection [8,9]. Therapeutic drug monitoring (TDM) of cyclosporine is therefore a well-established component of post-transplant care [10,11,12]. TDM of cyclosporine is typically performed by measuring whole blood trough concentrations (C0), although alternative sampling strategies such as 2 h post-dose concentrations (C2) have been used in selected clinical contexts. Target concentration ranges vary depending on transplant type, time since transplantation, immunological risk, and concomitant immunosuppressive therapy. Individualized dose adjustment based on measured concentrations is particularly important during the early post-transplant period and during changes in therapy.

Cyclosporine exhibits substantial interindividual and intraindividual pharmacokinetic variability. It is extensively metabolized by cytochrome P450 3A (CYP3A4 and CYP3A5) enzymes and is a substrate for P-glycoprotein transport [13,14,15]. Variability in intestinal absorption, hepatic metabolism, drug–drug interactions, and formulation differences contribute to nonlinear pharmacokinetics, in which small dose changes may lead to disproportionate changes in blood concentrations [14]. These characteristics make empirical dosing unreliable and underscore the necessity of TDM in routine clinical practice [16].

Given these analytical and pharmacokinetic challenges, this study aimed to evaluate long-term trends in patient median cyclosporine concentrations, assess potential temporal or seasonal variation, and compare patient medians with conventional statistical measures such as means and quartiles. The use of patient medians as a continuous quality indicator may provide additional insight into analytical stability and clinical practice patterns, particularly in settings where traditional quality control procedures are constrained by commutability limitations or restricted access to control materials.

## 2. Materials and Methods

### 2.1. Samples

Routine requests for cyclosporine blood concentration analysis at the Departments of Clinical Chemistry and Pharmacology, Akademiska Hospital, Uppsala, were included in the study. Whole blood samples were collected in EDTA tubes (367862, BD Vacutainer Systems, Plymouth, UK). The study period extended from 1 January 2006 to 31 December 2025. A total of 48,835 cyclosporine results were reported during this period. The samples came from 2027 individual patients tested over a time period of 20 years. Data were extracted in accordance with ethical approval and were fully pseudonymized. Available variables included date of sampling, patient age in years, sex, and measured cyclosporine concentration. No identifiable patient information was accessed. The study was approved by the Regional Ethical Review Board at Uppsala University (Dnr 01-367).

### 2.2. Instruments

Cyclosporine concentrations were initially measured using an immunoassay method on the Hitachi 912 analyzer (Roche Diagnostics, Rotkreuz, Switzerland) with reagents supplied by Microgenics (100147, Fremont, CA, USA). In February 2008, the method was transferred to the Architect platform (Abbott Laboratories, Abbott Park, IL, USA) using the same reagents supplied by Microgenics. Calibration and internal quality control were performed according to the manufacturer’s recommendations. External quality assessment was provided by UK NEQAS and analyzed on a monthly basis. The method coefficient of variation (CV) was 6.3% at 60 µg/L, 2.5% at 167 µg/L and 2.4% at 290 µg/L.

In January 2021, the cyclosporine assay was transferred from the Architect platform to the Cobas Pro e 801 analyzer (Roche Diagnostics) using cyclosporine reagents from the same manufacturer (07251246190, Roche Diagnostics). Prior to clinical implementation, the method transition was analytically validated according to local laboratory guidelines. Validation procedures included comparison of patient samples analyzed on both platforms, assessment of bias and imprecision, verification of calibration stability, and evaluation of analytical measuring range. The observed agreement between methods was deemed acceptable for clinical use, despite slightly lower values with the Roche method (approximately 7%).

External quality assessment was performed monthly using materials from LGC Standards (Wesel, Germany). The total CV for the method was 3.3% at 75 µg/L, 2.6% at 165 µg/L and 7.8% at 408 µg/L.

### 2.3. Statistical Calculations

Patient percentiles were calculated on a yearly basis from all reported cyclosporine results. Descriptive statistical analyses, including medians, quartiles, 10th, and 90th percentiles were used to examine longitudinal trends and variability over time. Statistical analyses were performed using Excel 365 (Microsoft Corp., Seattle, WA, USA) and Statistica version 10 (TIBCO Software, Palo Alto, CA, USA).

## 3. Results

### 3.1. Changes in Number of Reported Cyclosporine Results over Time

**Figure 1:**
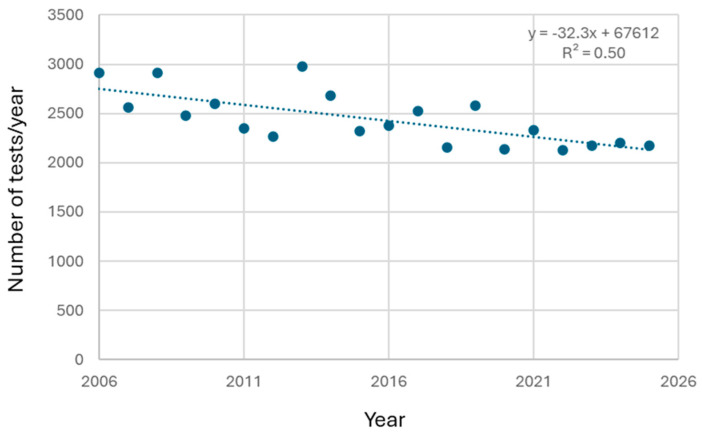
Number of reported cyclosporine results for each year 2006–2025.

The total number of requests during the study period was 48,835. Of these requests, 20,920 were for female patients and 28,011 were for male patients. A statistically significant negative trend was observed for the annual number of analyses over the 20-year period, indicating a decrease in the number of analyses over time (Figure 1).

The estimated slope corresponded to a mean reduction of 32.3 analyses per year (β = −32.3; 95% confidence interval −48.5 to −16.2; *p* = 0.0005) in the number of yearly reported results from 2917 requests in 2006 to 2177 requests in 2025.

### 3.2. Changes in Reported Cyclosporine Results over Time

**Figure 2:**
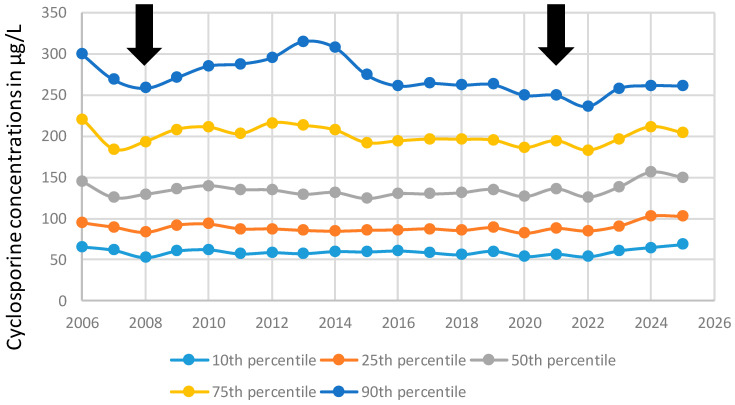
The 10th, 25th, 50th, 75th and 90th percentiles for each year 2006–2025. The arrows denote method changes in February 2008 and January 2021.

The median cyclosporine concentration was 134.4 µg/L with similar results for males and females (female patients 134.1 µg/L and male patients 134.5 µg/L) for the whole study period (Table 1 and Figure 2). There were very small gender differences for all studied percentiles from the 10th to the 90th percentile. The 10th, 25th, and 50th percentiles were stable during the studied time period 2006–2025 with total coefficient of variations (CV) between 6 and 7% (Table 2). For the highest percentile, the values fluctuated to a higher degree but still the total CV for yearly values was 7.6%.

### 3.3. Seasonal Variation in Cyclosporine Results

**Figure 3:**
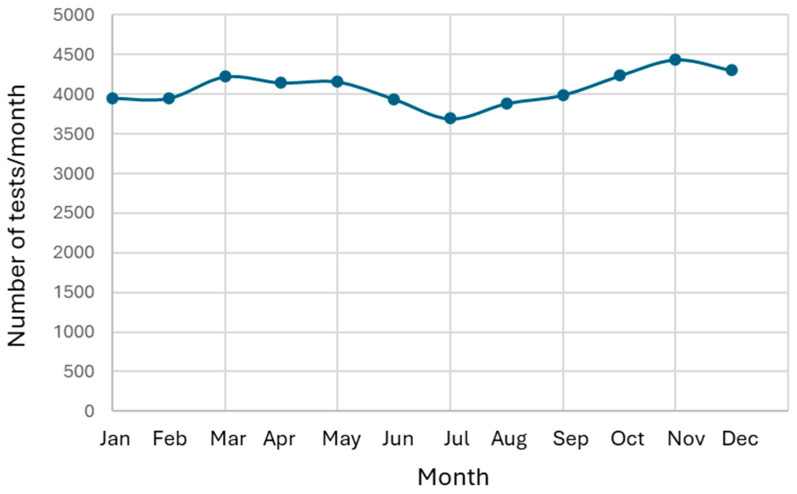
Number of reported cyclosporine results per month for the study period.

**Figure 4:**
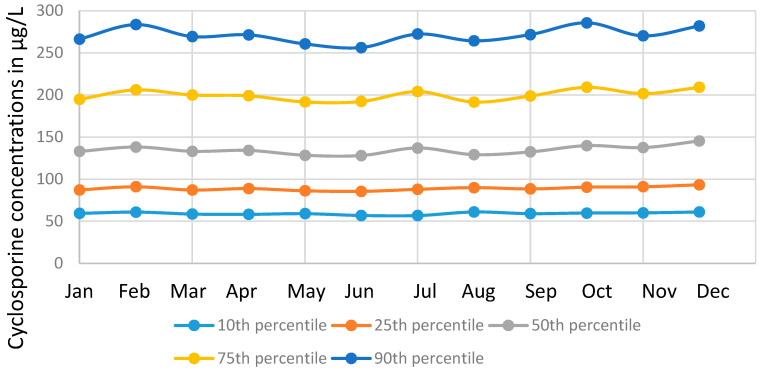
The 10th, 25th, 50th, 75th and 90th percentiles for individual months.

July is the main vacation month in Sweden. July was also the month with the lowest number of requests (Figure 3). Despite the variation in test requests, the cyclosporine results remained stable over the year (Figure 4).

### 3.4. Method Comparison Between the Architect and Cobas Cyclosporine Results

**Figure 5:**
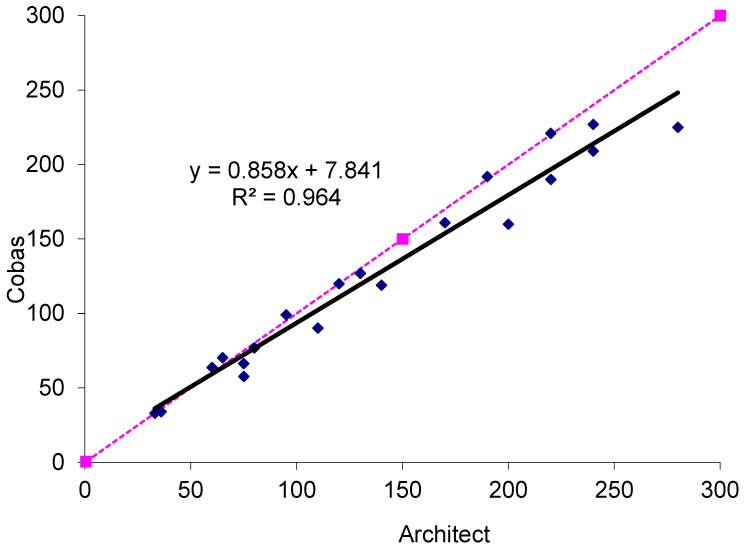
Comparison between the Architect and Cobas methods at the time of method change in January 2021. Blue squares represent individual patient sample results, each plotted as a paired measurement: Architect value on the x-axis and Cobas value on the y-axis. The black solid line is the linear regression line fitted to the patient sample data. It summarizes the relationship between the two methods and provides the regression equation and R^2^ value shown in the figure. The magenta dashed line represents the line of identity (y = x). It shows where the points would lie if the two methods produced identical results across the entire measuring range. The magenta squares at the ends mark the anchor points used to draw this reference line.

The method comparison consisted of 21 samples covering the measuring range of 30–227 µg/L. At 100 µg/L, the mean difference was 5.8% and increased to 8.4% at 250 µg/L (Figure 5).

We compared assay results from the two years preceding the method change (Architect) with those from the two years following the change (Cobas). The median cyclosporine concentration during the Architect period was 131.5 (95% confidence interval [CI] 128.7–134.5; *n* = 4809), whereas during the Cobas period it was 130.0 (95% CI 127.0–133.0; *n* = 4510). There were no statistically significant differences between results from the two study periods, as assessed by the Mann–Whitney test (*p* = 0.30).

## 4. Discussion

In this study, we evaluated long-term trends in routine cyclosporine blood concentrations over a 20-year period using patient-based statistical metrics. The principal finding is the remarkable stability of patient medians and lower percentiles, with minimal variability across time, sex, and analytical method transitions. These observations suggest a high degree of consistency in both analytical performance and clinical dosing practices.

The overall median cyclosporine concentration of approximately 134 µg/L remained essentially unchanged throughout the study period. This value aligns well with commonly applied trough concentration targets for maintenance immunosuppression in solid organ transplantation, particularly in kidney transplant recipients several months to years post-transplantation [17]. The absence of clinically meaningful differences between female and male patients across all assessed percentiles further supports the robustness of the observed trends at the population level.

Lower percentiles (10th and 25th percentiles) and the median showed the least variability over time, with coefficients of variation between 6% and 7%. These findings are notable given the substantial inter- and intraindividual pharmacokinetic variability of cyclosporine [18], as well as changes in transplant populations, immunosuppressive regimens, and clinical guidelines over two decades. The stability of these metrics suggests that patient-based statistics may be particularly effective in monitoring long-term analytical consistency and detecting gross shifts in clinical practice.

In contrast, higher percentiles demonstrated somewhat greater variability, which is not unexpected. Upper tail concentrations are more likely to reflect transient clinical scenarios, including early post-transplant periods, intentional dose escalation in high-immunological risk patients, drug–drug interactions, or episodes of non-adherence. Nevertheless, even the 90th percentile displayed a total coefficient of variation below 8%, indicating that extreme values did not increase progressively over time.

Importantly, no systematic changes in patient percentiles were observed following transitions between analytical platforms, despite a documented negative bias of approximately 7% with the Roche Cobas Pro method compared with the preceding Architect platform. This suggests that the calibration and validation procedures implemented during method transitions were sufficient to maintain continuity in reported patient results at the population level. Patient-median monitoring therefore appears to be resilient to moderate analytical bias, further supporting its utility as a longitudinal quality indicator.

The slight decline in the number of yearly requests likely reflects evolving clinical practices, including changes in transplant activity, the increasing use of alternative immunosuppressive agents such as tacrolimus, and potentially more individualized or less frequent monitoring strategies in stable long-term transplant recipients. However, this reduction did not appear to influence patient-level concentration distributions. The decline in annual test volume may partly be due to a shift from cyclosporine to tacrolimus as the number of reported tacrolimus results increased from 5616 in 2006 to 7320 in 2024 [19].

From a quality assurance perspective, patient-based real-time quality control metrics have gained increasing attention, particularly for assays where commutable control materials are limited or external quality assessment samples may not fully reflect patient matrix effects. The present data demonstrate that patient medians and percentile distributions can offer a powerful complementary approach to traditional internal and external quality control, especially for long-term retrospective surveillance.

Several limitations should be acknowledged. Patient-based medians reflect not only analytical performance but also clinical practice, sampling timing, dosing, interactions, and other factors. The study lacked detailed clinical information, including transplant type, time since transplantation, dosing information, sampling time relative to dosing, and concurrent medications. Consequently, clinical decision-making and patient-level outcomes could not be directly assessed. In addition, results from trough and non-trough samples could not be distinguished. The samples came from 2027 individual patients tested over a time period of 20 years. The total of 48,835 results corresponds to approximately 2450 results per year, which is slightly more than 1 test result per year and patient. The fact that patients contributed multiple results at different time points could cause a bias in the calculated annual percentiles. Nevertheless, the large sample size and extended observation period mitigate some of these limitations and allow robust population-level inference.

In conclusion, this study demonstrates long-term population-level stability of reported results over two decades of routine clinical practice. Monitoring patient medians and percentiles provides a simple, robust, and informative complement to conventional quality control strategies and may be particularly valuable for long-term assay surveillance in clinical laboratories.

## Figures and Tables

**Table 1:** Percentiles for the studied time period 2006–2025 for the whole study group and females and males.

	Total	Females	Males
10th percentile	59.1	59.1	59.0
25th percentile	88.6	88.9	88.4
50th percentile	134.4	134.1	134.5
75th percentile	200.0	199.5	200.7
90th percentile	271.1	272.7	270.4
Number	48,931	20,920	28,011

**Table 2:** Mean SD and coefficient of variations for the different percentiles for the time period 2006–2025.

	10th Percentile	25th Percentile	50th Percentile	75th Percentile	90th Percentile
Mean	59.37	89.09	134.40	199.90	271.38
SD	4.01	5.82	8.26	10.82	20.65
CV	6.76	6.53	6.15	5.41	7.61

## Data Availability

Under Swedish law, the authors cannot share the data used in this study and cannot conduct any further research other than what is specified in the ethical permissions application. For inquiries about the data, please contact the corresponding author with requests for and assistance with data. If the university approves the request, researchers can submit an application to the Regional Ethical Review Board for the specific research question that the researcher wants to examine.
